# Enhancing the Predictive Power of Google Trends Data Through Network Analysis: Infodemiology Study of COVID-19

**DOI:** 10.2196/42446

**Published:** 2023-09-07

**Authors:** Amanda MY Chu, Andy C Y Chong, Nick H T Lai, Agnes Tiwari, Mike K P So

**Affiliations:** 1 Department of Social Sciences and Policy Studies The Education University of Hong Kong Hong Kong Hong Kong; 2 School of Nursing Tung Wah College Hong Kong Hong Kong; 3 Department of Information Systems, Business Statistics and Operations Management The Hong Kong University of Science and Technology Hong Kong Hong Kong; 4 School of Nursing Hong Kong Sanatorium & Hospital Hong Kong Hong Kong; 5 Li Ka Shing Faculty of Medicine The University of Hong Kong Hong Kong Hong Kong

**Keywords:** internet search volumes, network analysis, pandemic risk, health care analytics, network connectedness, infodemiology, infoveillance, mobile phone, COVID-19

## Abstract

**Background:**

The COVID-19 outbreak has revealed a high demand for timely surveillance of pandemic developments. Google Trends (GT), which provides freely available search volume data, has been proven to be a reliable forecast and nowcast measure for public health issues. Previous studies have tended to use relative search volumes from GT directly to analyze associations and predict the progression of pandemic. However, GT’s normalization of the search volumes data and data retrieval restrictions affect the data resolution in reflecting the actual search behaviors, thus limiting the potential for using GT data to predict disease outbreaks.

**Objective:**

This study aimed to introduce a merged algorithm that helps recover the resolution and accuracy of the search volume data extracted from GT over long observation periods. In addition, this study also aimed to demonstrate the extended application of merged search volumes (MSVs) in combination of network analysis, via tracking the COVID-19 pandemic risk.

**Methods:**

We collected relative search volumes from GT and transformed them into MSVs using our proposed merged algorithm. The MSVs of the selected coronavirus-related keywords were compiled using the rolling window method. The correlations between the MSVs were calculated to form a dynamic network. The network statistics, including network density and the global clustering coefficients between the MSVs, were also calculated.

**Results:**

Our research findings suggested that although GT restricts the search data retrieval into weekly data points over a long period, our proposed approach could recover the daily search volume over the same investigation period to facilitate subsequent research analyses. In addition, the dynamic time warping diagrams show that the dynamic networks were capable of predicting the COVID-19 pandemic trends, in terms of the number of COVID-19 confirmed cases and severity risk scores.

**Conclusions:**

The innovative method for handling GT search data and the application of MSVs and network analysis to broaden the potential for GT data are useful for predicting the pandemic risk. Further investigation of the GT dynamic network can focus on noncommunicable diseases, health-related behaviors, and misinformation on the internet.

## Introduction

### Background

Since the discovery of the first case of COVID-19 in December 2019, the pandemic has continued to develop and spread globally for >3 years. With the new wave of COVID-19 that began in early 2022 owing to the Omicron variant [1], confirmed cases rose to 540 million and deaths rose to 6.3 million by the end of June 2022 [2]. To deal with such serious disease outbreaks, real-time disease surveillance plays a crucial role for policy makers in their efforts to effectively implement timely health measures and allocate resources. However, disease surveillance generally relies on laboratory testing results [3], which are limited by testing time and capacity [4]. Relevant clinical data, such as data for mortality, morbidity, and utility rate of the health system, are regarded as another layer of reliable population-scale indicators for tracking the spread of a pandemic [5]. Unfortunately, these official statistics usually lag behind the infection situation [4,6]. As a result, there is a need to explore other possible population-scale data as complementary sources for tracking pandemic risk in real time without the limitations of the traditional surveillance methods.

Internet use is a common human behavior worldwide, as reflected in the rapidly growing number of internet users and social media users globally [7]. In particular, the internet has become an important source of health care information [8]. Therefore, internet search data are now a valuable population-level resource for tracking disease development, complementing the traditional surveillance methods [9]. Consequently, an increasing number of infodemiological studies have sought to address the need for public health enhancement by applying internet user–contributed, health-related content. Investigations of infoveillance, an emerging type of public health syndromic surveillance that is based on information from the web [10], have been proposed for tracking various health issues [11-13].

Google Trends (GT) is a Google website that analyzes the popularity of search queries and thus provides a platform for the public’s use in retrieving the real-time search patterns of internet users worldwide [14]. As an official Google service, the aggregate GT data range from multiple sources of Google search engines, including web searches, image searches, news searches, Google shopping, and YouTube searches. There has been an upward trend in public health and epidemiological research on the use of GT in the last decade [15], and GT data have been proven to be reliable forecast and nowcast measures for official statistics and social topics, such as unemployment [16] and waves of pathogenic infections [17,18]. As the data in the repository are freely available and updated in real time, GT is considered to be a timely infoveillance tool that can complement the traditional surveillance methods well [9,19]. In particular, because the impact of an infectious disease will likely attract the public’s attention, many previous studies have attempted to investigate the associations between search volumes of internet queries and disease outbreak trends, such as with the outbreaks of Ebola [20] and Middle East respiratory syndrome coronavirus [21]. A methodology framework was also developed for working with GT data in infodemiology and infoveillance [22]. However, although web-based data are a good indicator for predicting changes in human behaviors [23], the application of GT data to health care research is relatively novel and has been evolving quickly only recently [24,25].

Although access to GT data is free, there are several limitations in retrieving the data. GT does not provide the actual number of searches for a particular topic of interest at a particular time and in a particular geographic region. Instead, it only shows “interest over time” by providing relative search volume (RSV) time series data, normalized to a time and geographic region, using a scale from 0 to 100. A value of 100 represents the peak popularity of a particular search interest at a particular time and location, and a value of 30 indicates that a particular search interest was 30% as popular as that of the peak search activity. Therefore, GT data over long time horizons will result in heavily normalized data with reduced resolution [18], thus rendering the gathered data noisy and limiting the predictive power of the data. The normalization of the GT data does offer convenience in comparison, but it also poses limitations in tracking search behaviors longitudinally with a high frequency, such as daily updating of search volumes. Early research on GT data also indicated that it is a nontrivial endeavor to aggregate information on GT data from multiple keywords. Therefore, there is a need to search for an effective method of retrieving GT data that increases the resolution of RSV to reflect a trend of interest and that integrates search data from multiple keywords.

Previous studies have tended to use the retrieved GT data of specified search queries for correlation analyses and modeling [9,11,19,23,26]. Many previous studies have been conducted on COVID-19–related keywords [18,27-30] and have demonstrated the importance of using multiple keywords. Although the inclusion of multiple search queries can help provide valuable information for tracking human behavior and public health issues [11], the linkage among the search queries remains a challenge for empirical studies seeking to analyze the data systematically [31]. Furthermore, we cannot ignore the potential of connectedness among search interests in reflecting the complexity of information searching behaviors among internet users. Understanding the complex search behavior will be crucial when using search data to develop an effective infoveillance system for infection outbreaks.

### Objectives

This study sought to extend the application of GT data in predicting disease outbreaks by demonstrating the application of merged internet search volumes and to propose a dynamic network approach to tracking COVID-19 pandemic risk. Specifically, we introduced the construction of GT networks over time, in which the network nodes were represented by different keywords. Our proposed GT network method can help incorporate internet search information from multiple keywords into a network. Using GT network structures and related network statistics, we were able to study the dependence among keywords—for example, COVID-19–related words—and learn topological features of search volumes of different keywords. We also examined the predictive power of the proposed application of our GT network method through trend analysis and dynamic time warping (DTW). In the case of COVID-19, which we studied in this research, we investigated the implications of topological features on the COVID-19 pandemic risk.

## Methods

This section describes the methodology used in this study. A set of custom Python scripts was used for data acquisition, cleansing, analysis, and most of the data visualization. *Gephi* 0.9.7 from Gephi Consortium [32] was used to create the base network graphs.

### Data Acquisition

In this study, the aggregated data from GT were retrieved for analysis. We used the time series data of “interest over time” as the RSV. The numerical data, ranging between 0 and 100, indicated the “search interest relative to the highest point” for the defined filtering region and the period. However, according to Google’s own definition, the value of “50” means the search term is “half as popular,” whereas “0” means low search volume was recorded for the term (ie, <1% of the searches attributed to the most searched term in the search list) [33]. This reflects that the “interest over time” can be regarded as a latent variable of the actual volume of searching. However, noise may be introduced through rounding and normalization. Although noise is unavoidable, we attempted to alleviate the effects of noise through a shingling technique, as proposed in the *Transforming Multiple RSVs Into a Single MSV* section, and to focus on the analysis of the constructed temporal network.

### Discovery of Related Keywords

Many previous studies used “related queries” to widen their scope of keyword coverage. Although this approach increases the number of search terms drastically, we opted to use “related topics” (RTs) because of their unambiguous characteristics and the inclusion of translations of the same concept into other languages.

For example, if a researcher is interested in understanding the search volume for Apple Inc, the computer and smartphone manufacturing company, when one enters only the word “apple,” GT will return the results for that exact word, without much interpretation. Therefore, the resulting data can hypothetically include a news report for the computer company’s latest product and the nutritional information for the fruit. Instead, researchers can search for a “topic” that filters out searches irrelevant to the computer manufacturer by picking “Apple (Technology Company).”

However, to the best of our knowledge, GT does not provide a public repository of the available topics. Yet, when a user enters certain keywords, GT will recommend a list of related queries and topics that can be used as our starting seed topics. On the basis of this method, we entered several queries, namely, “COVID-19” and “coronavirus,” which yields 3 RTs: “Coronavirus disease 2019 (Disease),” “Severe acute respiratory syndrome coronavirus 2 (Virus),” and “Coronavirus (Virus).” We continuously downloaded the RTs of the 3 seed topics we yielded in a 7-day sliding window from January 29, 2020, to March 4, 2022. All the keywords prompted by GT were included in the pool of keywords.

To remove the regional effects from our results, keywords related to specific regions were excluded. We determined that a term was region related if it was categorized by GT as one of the following: “country,” “state,” “province,” “prefecture,” “municipality,” “island,” “region,” “county,” “capital,” “city,” “autonomous community,” and “town.” For example, when we receive an RT “Stay-at-home order (Topic)” (machine tag: “/g/11hf9srvdz”) from GT, we will include it in our network, as it was determined as a “Topic” and is not in our exclusion category list. In contrast, if we receive an RT “Germany (Country in Europe)” (machine tag: “/m/0345h”) from GT, we will discard the topic, as the topic was categorized as “country” by Google, which is included in our exclusion category list.

After filtering, 228 topics were selected for this research, referred to as the topic set 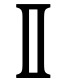 (see Textbox 1 for the search terms used).

**Selected keywords related to the COVID-19 pandemic, used in the construction of our temporal network.**

Coronavirus, Vaccine, COVID-19 vaccine, COVID-19 testing, Test, Signs and symptoms, Symptom, Symptoms of COVID-19, Strain, Severe acute respiratory syndrome coronavirus 2, Worldometers, Statistics, Variant, AZD1222, AstraZeneca, Nasal congestion, Mutation, Emmanuel Macron, Traffic light, GOV.UK, Bill, Preventive healthcare, Northern Beaches Council, Nueva Cepa, Certificate, Virus, Pfizer, BNT162b2, Side effect, Rapid antigen test, Bell's palsy, Transmission, Isolation, Ivermectin, Food and Drug Administration, mRNA-1273, Centers for Disease Control and Prevention, COVID-19 pandemic in New York City, Polymerase chain reaction, Guideline, British Columbia, CVS Pharmacy, Drive-through, CVS Health, Mink, Regions of Italy, Galicia, TousAntiCovid, contagium, Tier 1 network, Blood type, Tier 2 network, Nasopharyngeal swab, 2020 coronavirus pandemic in Scotland, Incubation period, Case, Donald Trump, 2019–20 coronavirus pandemic related application, NHS COVID-19, National Health Service, Dashboard, Common cold, Silvio Berlusconi, Pandemic, Medical test, Departments of France, Serology, Infection prevention and control, Swine influenza, Herman Cain, Hydroxychloroquine, Provinces of Vietnam, COVID-19 pandemic in Victoria, Sore throat, University of Oxford, COVID-19 party, Jair Bolsonaro, World Health Organization, Airborne transmission, 2020 coronavirus pandemic in Canada, Worldometer COVID-19 Dashboard, County, COVID-19 pandemic in Texas, Asymptomatic, Disease outbreak, Rapid diagnostic test, Johns Hopkins University, Mortality rate, Antibody, Maharashtra Police, DG Police Office Mumbai, COVID-19 pandemic in West Bengal expected, Oise, Lysol, Infection, 2019–20 coronavirus pandemic, Death, Chinese language, Influenza, Pneumonia, China virus, Cruise ship, Cure, 2020 coronavirus pandemic in Singapore, Pangolins, Coronavirus disease 2019, SARS, Antigen, Immunoglobulin G, Variety, Prefectures of Japan, Coronavirus Alpha variant, Immunity, RNA, RNA virus, Peplomer, Roche Holding AG, Severe acute respiratory syndrome coronavirus, Roche, Disease cluster, Genome, Yahoo Japan Corporation, Robert Koch Institute, Medical laboratory, Ebola, Avian influenza, Breaking news, Nucleic acid test, NHK, Municipalities of Japan, Federal Office of Public Health of the Swiss Confederation, Test method, Immune system, Immunoglobulin M, Falling Number, Südwestrundfunk, interactive map, Subsidy, Remdesivir, Microscope, Molecular biology, Study group, Oct-04, Bulletin board system, Typhoon, Host, Lockdown, DNA, Hoax, Risk, Dwayne Johnson, 爆サイ, Assay, Shinzo Abe, South African Revenue Service, PTT Bulletin Board System, Reverse transcription polymerase chain reaction, Severance package, Health facility, Dabie bandavirus, Sequela, Inpatient care, Health professional, Go To Campaign, COVID-19 Contact-Confirming Application, Jul-02, U.S. state, Middle East respiratory syndrome–related coronavirus, Chemical structure, Basic reproduction number, Micrometre, HIV/AIDS, HIV, Accuracy and precision, Potency, Icelandic language, Interactivity, Wind wave, SARS outbreak, Genetics, Infectious disease, Bats, Patent, MERS, Spanish flu, Tasuku Honjo, Robert Koch, Kumiko Okae, Ministry of Health, Labour and Welfare of Japan, Website about COVID-19 pandemic in Vietnam, temporarily closed school, Case fatality rate, Mask, event, biological weapon, Diamond Princess, Vaccination rule, Point-of-care testing, Stay-at-home order ordinance, Incidence, Tagesschau, Districts of Germany, hot spot, Press conference, Podkarpackie Voivodeship, Dashboard, National Institute for Public Health and the Environment, Upper Austria, Child benefit, Demonstration, Travel warning, COVID-19 pandemic in Aichi prefecture, Amitabh Bachchan, Tönnies Holding, Patanjali Ayurved, Mayu Watanabe, Logistics, Public transport, Hygiene, Public health, Emergency medical services, Statistic, Advice, and News ticker

### Downloading Search Volume Data From GT

To systematically retrieve data from GT, we used the library *pytrends* [34]. We executed the custom script regularly to collect the RSV data. Each request that was sent gathered a set of 30-day “interest over time” data 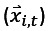 :



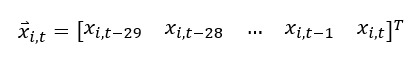



For example, the RSV data gathered for the “Coronavirus (virus)” from January 1, 2020, to January 30, 2020 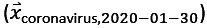 were:



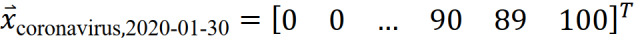



We then repeated the requests to collect data consecutively. We list all the terms in Textbox 1 (topic set 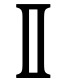). Our data collection period for this research was from January 1, 2020 (*t*= 2020-01-30) to January 31, 2022 (*t*= 2022-01-31).

### Merging of Multiple Short Time Series Trends Into a Glandular Time Series Over a Longer Period

Inspired by Park et al [35], we devised a range-based merging algorithm.

Let 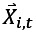 be an *n*-day actual search volume time series for a topic 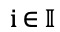 on the days lying in the interval 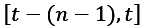, and let *X_i,t_* be the actual search volume for a topic *i* on date *t*:



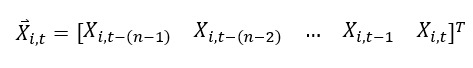



Assuming that GT applied the normalization with a common correction factor *C_t_* to the entire actual search volume time series, the RSV returned can be considered as 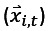:



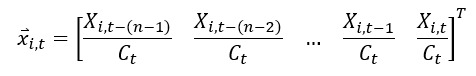



For any 2 consecutive GT RSV data sets, 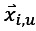 and 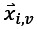, where *v = u + 1* and have the same data collection duration of *n* days, based on our previous assumption, 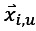 and 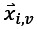 are normalized with the correction factors *C_u_* and *C_v_* within the interval [*u* − (*n* − 1), *u*] and [*v* − (*n* − 1), *v*], respectively.

Given *X*_(*i*,*v*)_ = *X*_(*i*,*u*+*1*)_:



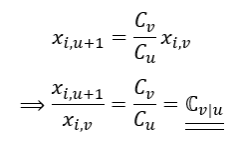



where



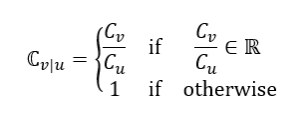



Through the moment estimator of 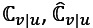, we can subsequently obtain the estimate of 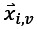 with respect to the RSV 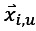, 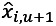:



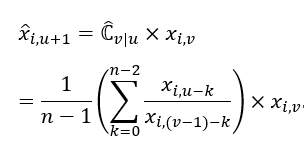



For example, considering the correction factor of the data point for the term “Coronavirus (virus)” on January 31, 2020, from 2 RSV series of n=30 is:



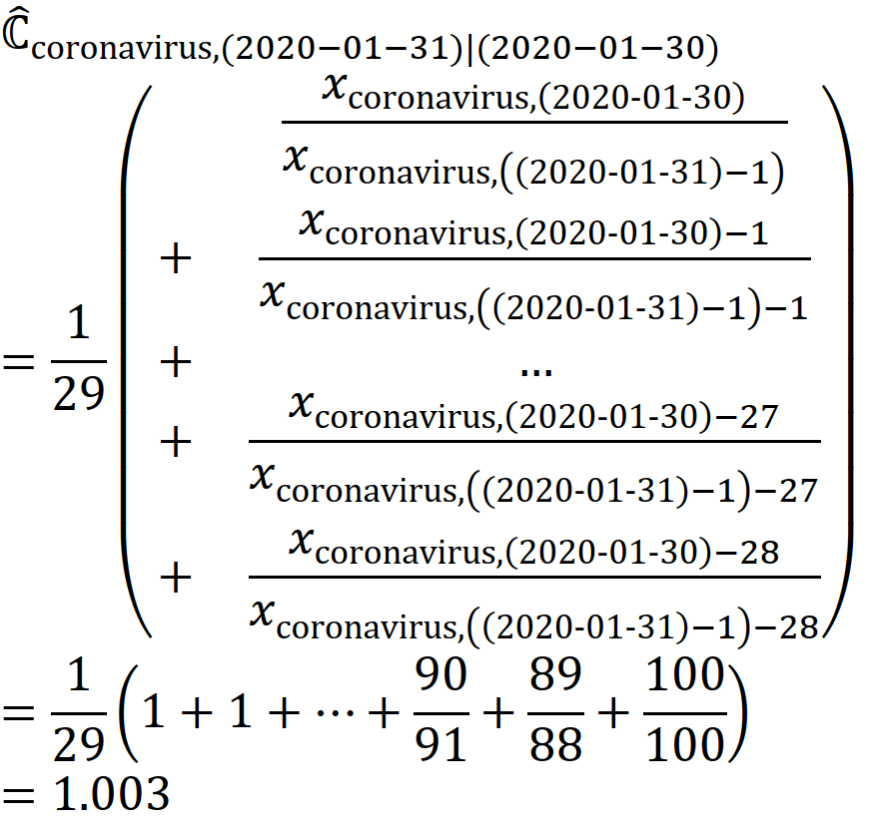



Thus, the adjusted data point for “Coronavirus (virus)” on January 31, 2020, is as follows:



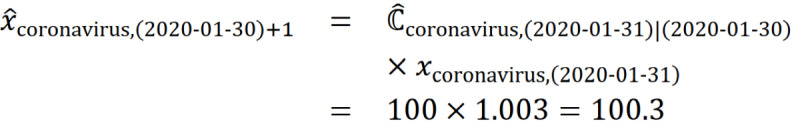



We can thus obtain a 31-day merged search volume (MSV) for “Coronavirus (virus),” as shown in Table 1.

Through multiple iterations of estimations using a total of *p* RSV time series, as shown in Tables 2 and 3, we can finally obtain an MSV time series 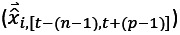 for the search term 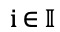 from *t* − (*n* − 1) to *t* + (*p* − 1):



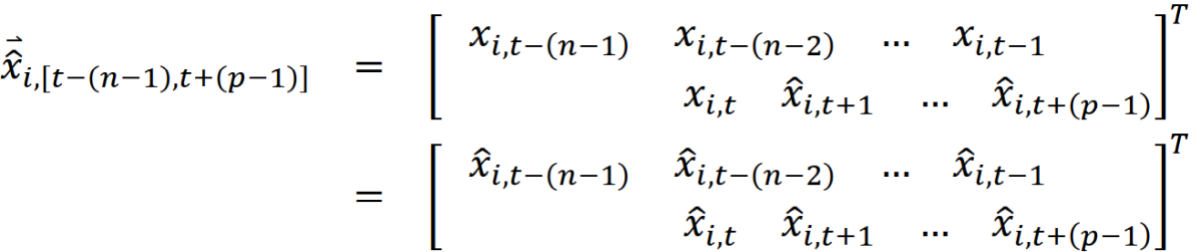



Continuing from our previous example, we can eventually obtain the MSV time series for “Coronavirus (virus)” as follows:



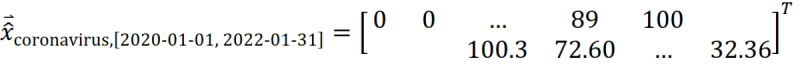



In this study, we opted to use RSV time series data with a length of 30 days (n=30), and the RSV time series for January 30, 2020, was used as the baseline against which other subsequent RSV series were calibrated.

**Table 1:** A sample calculation of the correction factor and adjusted data point of January 31, 2020 
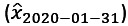
, using data sets from January 1, 2020, to January 30, 2020, and from January 2, 2020, to January 31, 2020. Data set keyword: “Coronavirus.”

Date	Data downloaded on January 30, 2020 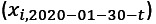	Data downloaded on January 31, 2020 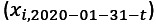	Correction factor for new data point on January 31, 2020 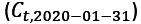	Merged Google Trends time series
January 1, 2020	0	Not available in the sequence	Out of scope	0
January 2, 2020	0	0	0 / 0 = 1.000	0
January 3, 2020	0	0	0 / 0 = 1.000	0
...	...	...	...	...
January 27, 2020	75	78	75 / 78 = 0.962	75
January 28, 2020	90	91	90 / 91 = 0.989	90
January 29, 2020	89	88	89 / 88 = 1.011	89
January 30, 2020	100	100	100 / 100 = 1.000	100
January 31, 2020	Not available in the sequence	100	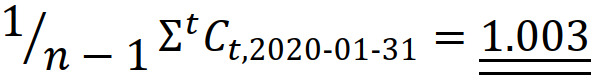	100 × 1.003 = *100.3*

**Table 2:** A sample calculation of the correction factor and adjusted data point of February 1, 2020 
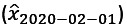
, using data sets from January 2, 2020, to January 31, 2020, and from January 3, 2020, to February 1, 2020. Data set keyword: “Coronavirus.”

Date	Data downloaded on January 31, 2020 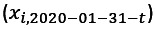	Data downloaded on February 1, 2020 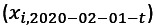	Correction factor for new data point on February 1, 2020 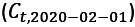	Merged Google Trends time series
January 2, 2020	0	Not available in the sequence	Out of scope	0
January 3, 2020	0	0	0 / 0 = 1.000	0
...	...	...	...	...
January 27, 2020	78	77	78 / 77 = 1.013	75
January 28, 2020	91	91	91 / 91 = 1.000	90
January 29, 2020	88	89	88 / 89 = 0.989	89
January 30, 2020	100	95	100 / 95 = 1.053	100
January 31, 2020	100	100	100 / 100 = 1.000	100 × 1.003 = 100.3
February 1, 2020	Not available in the sequence	72	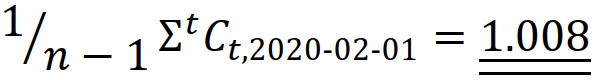	72 × 1.008 = *72.60*

**Table 3:** A sample calculation of the correction factor and adjusted data point of February 1, 2022 
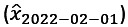
, using data sets from January 1, 2022, to January 30, 2022, and from January 2, 2022, to January 21, 2022. Data set keyword: “Coronavirus.”

Date	Data downloaded on January 30, 2022 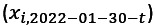	Data downloaded on January 31, 2022 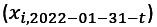	Correction factor for new data point on January 31, 2022 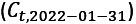	Merged Google Trends time series
January 1, 2022	55	Not available in the sequence	Out of scope	76 × 0.3007 = 22.85
January 2, 2022	64	60	24.88 / 60 = 0.4146	90 × 0.2764 = 24.88
...	...	...	...	...
January 26, 2022	94	90	38.17 / 90 = 0.4241	95 × 0.4018 = 38.17
January 27, 2022	89	87	36.14 / 87 = 0.4154	88 × 0.4107 = 36.14
January 28, 2022	86	82	33.87 / 82 = 0.4130	82 × 0.4107 = 36.14
January 29, 2022	83	77	32.08 / 77 = 0.4167	80 × 0.4010 = 33.87
January 30, 2022	79	76	31.77 / 76 = 0.4181	79 × 0.4022 = 31.77
January 31, 2022	Not available in the sequence	78	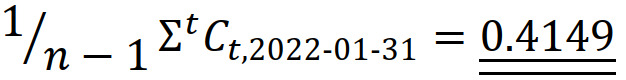	78 × 0.4149 = *32.36*

### Construction of Daily Search Volume Networks Using Merged GT Time Series Data

Let *N* be the size of the search term set 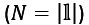. We considered the temporal network construction in an *R*-day rolling window. To better focus on the changes of MSVs and to ease the magnitude of differences between MSVs, we followed the studies by Chu et al [36] and So et al [37] to obtain square-rooted-differenced data. Thus, the square-rooted-differenced MSV matrix (**Y**_*t*_) at time *t* is as follows:



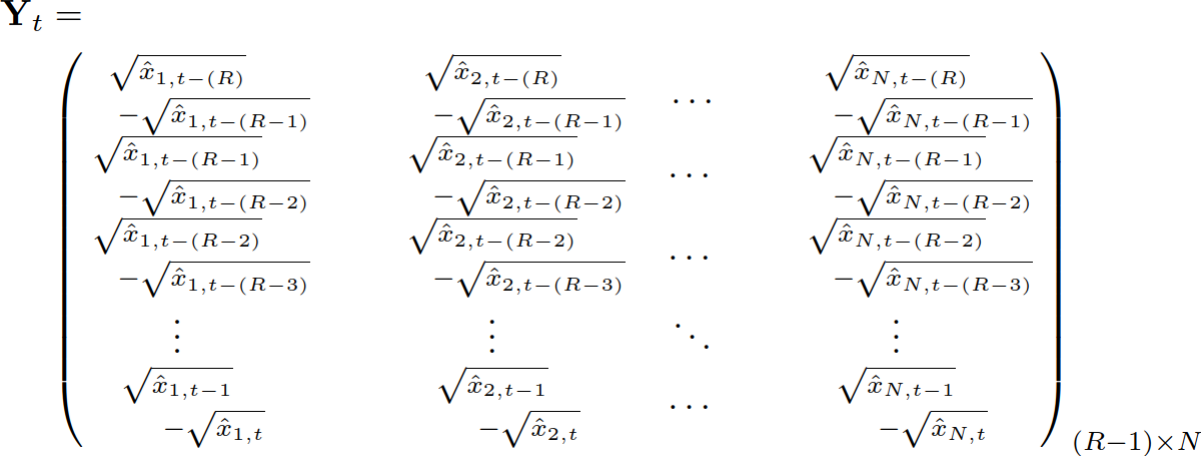



A covariance matrix (**ρ**_*t*_) of each search term in the square-rooted-differenced MSV matrix (**Y**_*t*_) can be determined, where the correlation between the search terms *i* and *j* at time *t* is 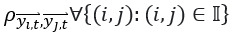.



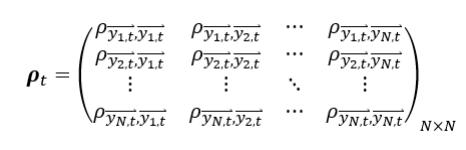



Using the correlation matrix **ρ**_*t*_, we can then construct an undirected network of search terms with an adjacency matrix **A**_*t*_ formation at time *t* as:



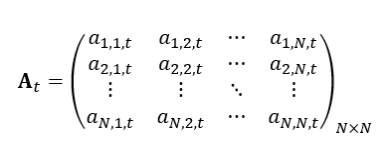





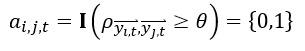



In this study, we used a rolling window length (*R*) of 30 days to construct the network.

### Calculation of Network Statistics

#### Network Density

On the basis of the method proposed by Chu et al [36], we defined the network density (*D_t_*) of a network at time *t* as follows:



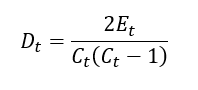



where *E_t_* is the number of edges in the network at time *t* and *C_t_* is the number of possible connections between all nodes (ie, search terms) at time *t*. *D_t_* assesses the connectedness of the nodes present at time *t*. Higher values represent higher connectedness at a given moment.

#### Global Clustering Coefficient

As defined by So et al [37], the global clustering coefficient (*GC_t_*) was calculated based on the local clustering coefficients *c_i,t_* of all nodes in the network at the time (ie, 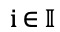). We first defined the calculation of the local clustering coefficients for the search term *i* as follows:



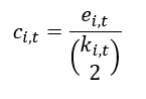



where *e_i,t_* is the “number of connected pairs among the neighbors of vertex *i* at time *t*” and *k_i,t_* is the “number of neighbors for each vertex.”

Thus, the global clustering coefficient (*GC_t_*) can be calculated as follows:



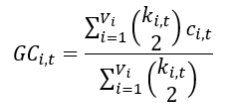



*GC_t_* represents the ratio of the number of established connections (edges) to the number of possible connections. The higher the value of the coefficient, the more interconnected the network nodes are.

#### Calculation of DTW

DTW is an algorithm for measuring the similarity between 2 time series by determining the shortest total distances between the 2 series [38]. It is useful to detect the leader-follower interaction. We used the Python library *dtw-python* (version 1.1.12) to create the DTW-aligned plots [39]. The algorithm in the library associates a node from the query time series (in our case, network statistics, network density, and global clustering coefficients) to a node from the template time series (confirmed COVID-19 cases in and severity risk score [SRS]) with the lowest distances.

#### Calculation of DTW Metric

To quantify the DTW results, we adopted the DTW-based metric proposed by Laperre et al [40]. The proposed algorithm augments the existing DTW algorithm by including a “forward-looking only” constraint, that is, a latent variable (such as network density and global clustering coefficient) observed at time *t + p* can only be mapped to a historical or the current observation (such as number of COVID-19 cases or SRSs) from *t* to *t + p*, where the time shift value 
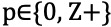
. A time shift value of 0 indicates that the latent variable moves in sync with the observation, whereas a value >0 indicates a leading effect of the latent variable on the observation.

#### Calculation of the SRS

Adopted from the study by So et al [37], the SRS represents the presymptomatic transmission owing to possible interaction between the susceptible population in one country and currently infected cases in another country, before the confirmed cases are identified and force isolated or quarantined. Higher scores indicate stronger signals that the pandemic is uncontrolled, which could be regarded as a systematic risk assessment.

### Ethical Considerations

Ethics approval is not required as no individually identifiable information was gathered, processed, or analyzed in this research.

## Results

### Transforming Multiple RSVs Into a Single MSV

To extract a search volume time series to reflect the trend of a specific search interest, we adopted a data transformation algorithm to construct MSVs using the rolling window approach. The primary idea was to produce a long time series of MSVs that could serve as a proxy for the search volume of keywords. Thus, the MSV series can be used to draw statistical inferences and predictions. MSV was produced by aggregating the RSV data while also exhibiting the trend indicated by the RSVs. With MSV, we were able to extend the investigation horizon beyond 9 months for daily observations and beyond 270 weeks for weekly observations, which was of interest because 9 months and 270 weeks are the longest periods for retrieving daily and weekly RSV data, respectively, from GT. Therefore, MSVs could be very helpful for tracking search interests over a long period in public health studies, such as in following the status of the COVID-19 pandemic, which has now lasted for >2.5 years.

We focused on “interest over time” as the proxy for the actual search volumes in Google. In this study, we did not specify any geographic regions but instead adopted the global web search data to obtain a more generic and globally inclusive searching phenomenon. Using these filter settings, the daily RSV data were extracted from GT for further data transformation, following the calculation procedure shown in Tables 1-3. Using the ratio of the “interest over time” data points for the same date in the 2 consecutive sequences, we calculated the correction factors for each day that existed in both sequences (as tabulated in the fourth column in the tables) to obtain the averages of the factors (as shown in the last cell in the fourth columns). The newly discovered data points (in this example, the data points on January 31, 2020; February 1, 2020; and January 31, 2022, respectively) were multiplied by the appropriate average correction factors (in this example, 1.003, 1.008, and 0.4149, respectively) to obtain the adjusted data point in the merged series. This process was repeated consecutively, as shown in Tables 1-3.

**Figure 1:**
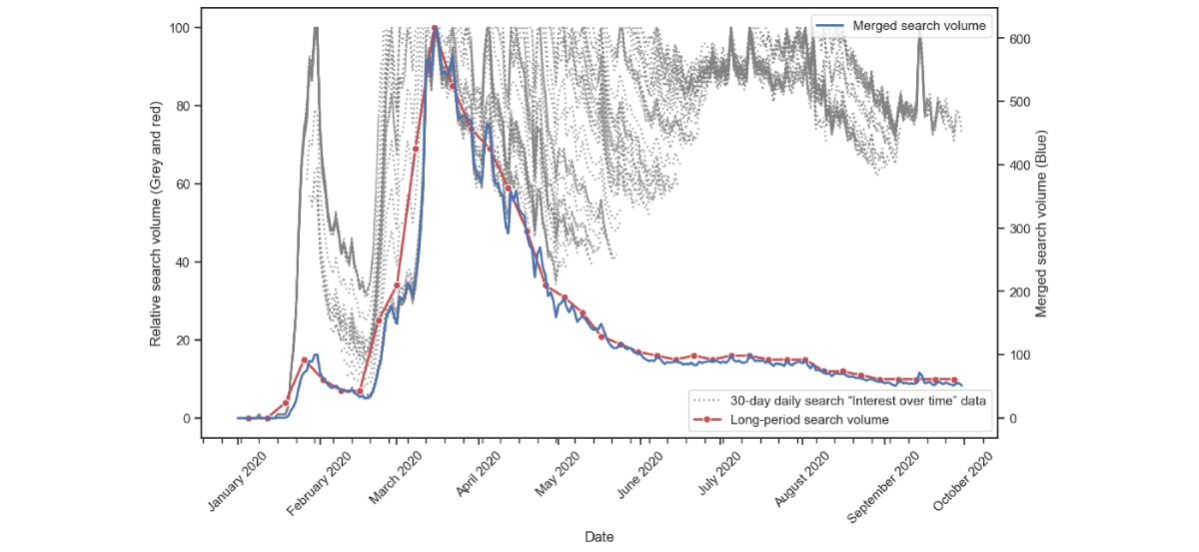
An example of the process of creating a merged search volume series (data set keyword: “Coronavirus”; dates: from January 31, 2020, to September 30, 2020). Dotted lines: the 30-day “interest over time” relative search volume (RSV) data set from Google Trends (GT). Red line: the weekly RSV series retrieved from GT. Blue line: the daily merged search volume series using our transformation algorithm to merge RSVs through a rolling window approach.

We further visualized and herein presented the process of creating the MSV series in Figure 1. Using the GT RSV data of the COVID-19 pandemic during the first 9 months as a demonstration, the 30-day “interest over time” data set (shown as 245 dotted lines) for the dates between January 1, 2020, and September 30, 2020, was downloaded from GT. We then calculated the next-day interest based on the data from the previous 29-day data and replicated the steps to compile the final MSV series (shown as the blue line in Figure 1) with 274 data points of daily MSV. The daily RSVs shown in the 245 dotted lines produced the relative search interest over a fixed period of 30 days. Extending the investigation period to longer than 30 days could reduce the sensitivity of the daily RSV in reflecting the daily time trend of the search interest. In contrast, the data directly retrieved from GT between January 1, 2020, and September 30, 2020, contained only 39 data points of weekly RSVs (shown by the red line in Figure 1). This reduction of data points implied that we needed to lower the time interval of investigation to 1 week, meaning that we could not study a daily dynamic in the search interest. Owing to the constraints of GT, we could only retrieve weekly RSVs of an interval of at most 270 weeks. Using our proposed MSV approach, we merged the 733 RSV series into one singular, extended MSV time series, aiming to recover the daily trend of the search interest. In fact, the patterns of the MSV (the blue line) and the weekly RSV (the red line) were similar, indicating that the transformation algorithm for constructing the MSV was reasonable in the sense that the trend of the search interest revealed by the MSV was consistent with the trend of the search interest revealed by the weekly RSV provided by GT. This example shows that although GT restricts the search data retrieval into weekly data points over a long period, our proposed approach could recover the daily search volume over the same investigation period to facilitate subsequent research analyses.

### Selection of Coronavirus-Related Search Keywords

To understand the associations between the search interest and the pandemic spread, we explored a list of coronavirus-related keywords based on the metrics of “related topic” provided by GT. We searched the related keywords for 3 seed topics: “Coronavirus disease 2019,” “Severe acute respiratory syndrome coronavirus 2,” and “Coronavirus,” with a 7-day sliding window.

By combining the shorter lists of keywords returned by GT when searching for the 3 seed terms in each 7-day period over 108 weeks (from January 1, 2020, to January 31, 2022), we obtained a raw list of keywords. Then, we filtered out the duplicates and irrelevant keywords and eventually settled on a keyword set of 228 elements (search topics), which are listed in Textbox 1. Using the transformation algorithm proposed in the *Transforming Multiple RSVs Into a Single MSV* section, the MSV of each keyword was calculated for dynamic network analysis, which involved compiling network statistics using related keywords as network nodes for a pandemic risk assessment.

### MSVs and COVID-19 Waves

**Figure 2:**
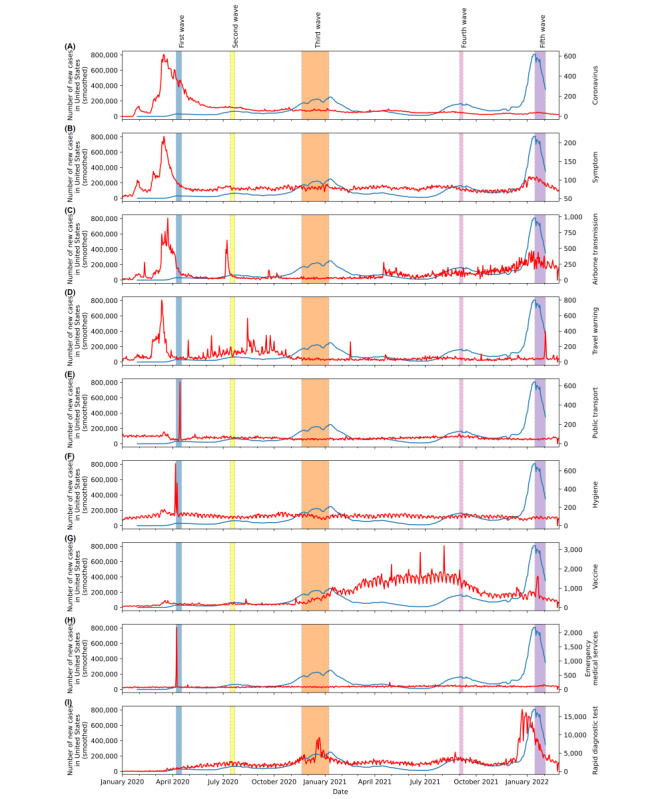
Comparison of US-confirmed new cases (blue line) sourced from the World Health Organization and the merged search volume of selected keywords, including (A) Coronavirus, (B) Symptom, (C) Airborne transmission, (D) Travel warning, (E) Public transport, (F) Hygiene, (G) Vaccine, (H) Emergency medical services, and (I) Rapid diagnostic test (red line), constructed by transforming the relative search volume of Google Trends from January 1, 2020, to February 3, 2022. Highlighted areas: the 5 wave peaks observed in confirmed new cases in the United States.

**Figure 3:**
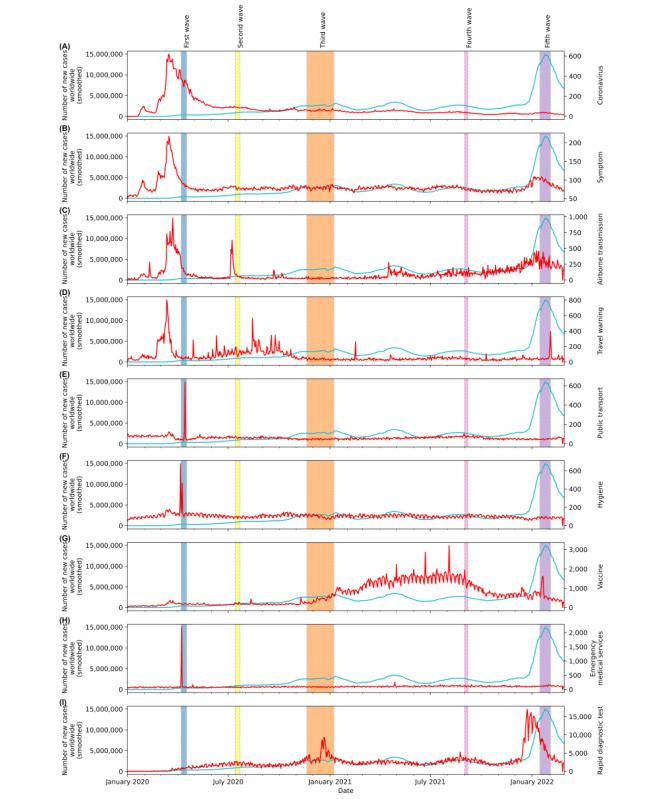
Comparison of worldwide confirmed new cases (cyan line) sourced from the World Health Organization and the merged search volume of selected keywords, including (A) Coronavirus, (B) Symptom, (C) Airborne transmission, (D) Travel warning, (E) Public transport, (F) Hygiene, (G) Vaccine, (H) Emergency medical services, and (I) Rapid diagnostic test (red line), constructed by transforming the relative search volume of Google Trends from January 1, 2020, to February 3, 2022. Highlighted areas: the 5 wave peaks observed in confirmed new cases in the United States.

To understand the relationship between internet search interest and pandemic development, the MSV trends of several selected keywords were compared in parallel with the newly confirmed COVID-19 cases (Figures 2 and 3). Owing to the severity of the COVID-19 pandemic in the United States compared with other countries, we evaluated the MSV data against the new COVID-19 cases in the United States in Figure 2, in addition to the global cases, as shown in Figure 3. We noticed a similar pattern between MSV data and the number of COVID-19 cases in the United States and globally. Therefore, we used the situation with COVID-19 in the United States as a basis for further discussion, and the peaks of the 5 waves of COVID-19 in the United States were highlighted in different colors as a reference to illustrate the different stages in the development of the pandemic. Diverse associations with the waves can be observed in the trend comparisons. For example, drastic reactions in MSVs can be observed for the keywords “Coronavirus,” “Symptom,” “Airborne transmission,” “Travel warning,” “Hygiene,” and “Emergency medical service” in Figures 2A-2D, 2F, and 2H before the number of COVID-19 cases reached the first wave peak (highlighted in light blue) in early April 2020. These abrupt increases in the search interest of the general pandemic-related keywords provide evidence of the capabilities of MSVs in the early detection of outbreak risks.

In contrast, the MSV trends for “Vaccine” and “Rapid diagnostic test” in Figures 2G and 2I show different variations during the latter waves of the COVID-19 pandemic. Although vaccination and efficient diagnostic tests are typically developed in the later phase of an emerging infectious disease, the corresponding MSV trends still aligned with symbolic milestones in COVID-19 control efforts. This occurred when effective rapid diagnostic tests and vaccines for COVID-19 became available in the United States, during and after the third pandemic waves, respectively. These diverse associations between the search interest of keywords and pandemic developments also motivated and supported our approach to investigating the dynamic connectedness among the coronavirus-related search keywords through network analysis.

We also compared the MSV trends of the selected keywords with the number of confirmed new COVID-19 cases worldwide in Figure 3. In Figure 3, we can draw similar conclusions to Figure 2. We also noted an increase in confirmed cases of COVID-19 globally from April to May 2021, which correlated with the sharp rise in the MSVs of “Airborne transmission” and “Vaccine.”

### Dynamic GT Network Construction

After computing the MSVs of all the selected keywords, we constructed a dynamic GT network based on the correlations between the search volume data of pairs of selected keywords. In the network analysis terminology, we established an edge (a connection) between 2 keywords (also known as nodes in a network) if the corresponding correlation coefficients were significantly high (when the correlation coefficient is >0.5; the threshold we adopted) [36,37]. This approach allowed us to quantify the connectedness of the relevant search queries using temporal dynamic network statistics.

As the search volume of keywords from a common topic (COVID-19, in our case) represents people’s concerns about the topic, we anticipated that any common trend or comovement of the COVID-19–related keywords could indicate people’s interest in the pandemic as they looked for relevant helpful information through internet searches. An increase in the MSVs of different keywords simultaneously might indicate an increasing concern about the common topic associated with the keywords.

To incorporate common trends in the search interest of multiple keywords, network analysis is a viable approach [41-45]. A network is a natural configuration that integrates common trends of information about multiple keywords together, from which we can perform a network analysis; for example, based on network statistics, we can understand the general interest in a collection of keywords simultaneously. Through the connectedness of a GT network [43,46-48], we quantified the coherence of changes in search interest for different keywords over time and studied the implications of the pandemic risk in this research. The set of network nodes is represented by the set of keywords, which is constant over time. However, the set of edges changes over time to reflect time-varying patterns of search interests. By construction, the MSV represents a proxy time series of the search volume, and the correlation of changes in the MSVs of 2 keywords exceeding a certain threshold can be interpreted as a strong comovement whose occurrence can be used to define an edge of the 2 keywords. For example, if the correlation between changes in the MSV of “Coronavirus” and “Symptom” at time *t* is >0.5 (the correlation threshold we took in this research), we created an edge between the nodes “Coronavirus” and “Symptom” in the GT network at time *t*. This construction mechanism used the same rationale that was used in the pandemic network constructions in other studies [43,46,49]; however, instead of using the number of confirmed COVID-19 cases in regions, we used the MSVs of keywords.

**Figure 4:**
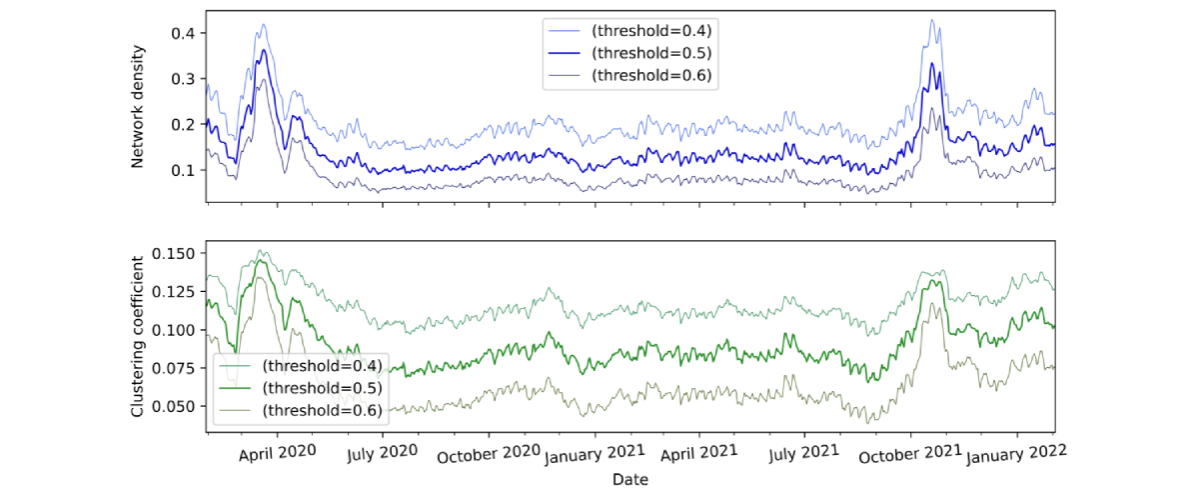
A comparison of network density and clustering coefficient constructed by 3 different correlation thresholds at 0.4, 0.5 (the threshold used in the study) and 0.6.

In addition to using 0.5 as the correlation threshold, we used 2 alternating correlation thresholds—0.4 and 0.6 for sensitivity analysis. A comparison of the network density and the global clustering coefficient constructed by the 3 different correlation thresholds at 0.4, 0.5 (the threshold used in this study), and 0.6 is shown in Figure 4. The pattern of the network density plots with thresholds of 0.4 and 0.6 is very similar to that of the one with a threshold of 0.5. We also observed a similar phenomenon in the global clustering coefficient, with an exception in March 2020, where the coefficient with a threshold of 0.6 showed a marginally narrower peak than those with thresholds of 0.4 and 0.5.

Following the studies by Chu et al [36], So et al [37], and So et al [46], the correlations of changes in MSV at time *t* were calculated using a rolling window approach that was based on the past *R*-day (including time *t*) MSV changes of keywords; therefore, the topology of the edges in the GT network would reflect the search interest characteristics of multiple keywords at time *t*. Ultimately, we had a constellation of dynamic GT networks constructed by correlations of changes in MSVs among keywords. Experience from the early research papers on COVID-19 revealed that the dynamic GT network, which summarized the infodemiology of relevant keywords, could provide an effective visualization of a pandemic situation [46], early warning signals of the pandemic [36], and pandemic risk assessments [37,50].

**Figure 5:**
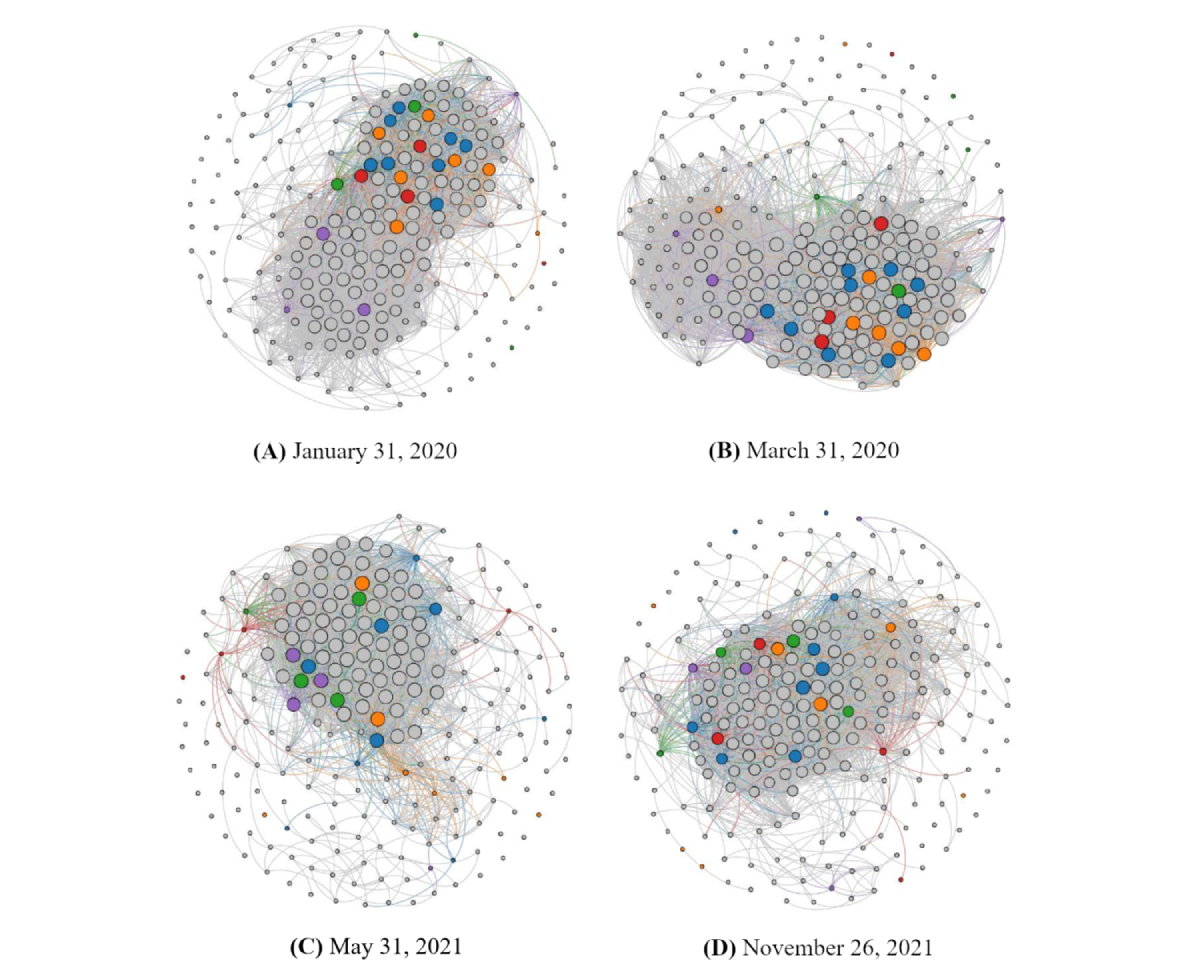
Snapshots of the dynamic Google Trends network. Snapshots were taken of the network on (A) January 31, 2020, (B) March 11, 2020, (C) April 31, 2021, and (D) November 26, 2021. Nodes are highlighted with different colors to represent the top 5 categories: diseases (blue), viruses (orange), vaccines (green), government agencies (red), and pharmaceutical companies (purple).

Figure 5 depicts a snapshot of the dynamic GT networks for 4 significant events: “Public Health Emergency of International Concern” on January 31, 2020, “COVID-19 was declared as a global pandemic by WHO” on March 11, 2020, “the Delta variant was named” on May 21, 2021, and “the Omicron variant was named” on November 26, 2021. Figure 5 also highlights the nodes with the top 5 most frequently appearing topic types: diseases (blue nodes), viruses (orange), vaccines (green), government agencies (red), and pharmaceutical companies (purple). It is obvious that the nodes related to diseases (blue nodes) occurred in the central cluster in all 4 snapshots. Several virus-related nodes (orange nodes), such as another official name for COVID-19 (“severe acute respiratory syndrome coronavirus 2” or “SARS-CoV-2”), were also included in the central clusters in most of the 4 time points we studied. These 2 observations matched our intuition that the general public seeks unknown information about COVID-19 through the search engine.

It was found that the vaccine-related nodes (green nodes) had a high degree of connectedness on all sampling days, even before any COVID-19 vaccine was approved for administration. In Figures 5C and 5D, disease-related and virus-related nodes drifted away from the cluster center, whereas nodes related to vaccines and pharmaceutical industry companies moved toward the center of the cluster.

**Figure 6:**
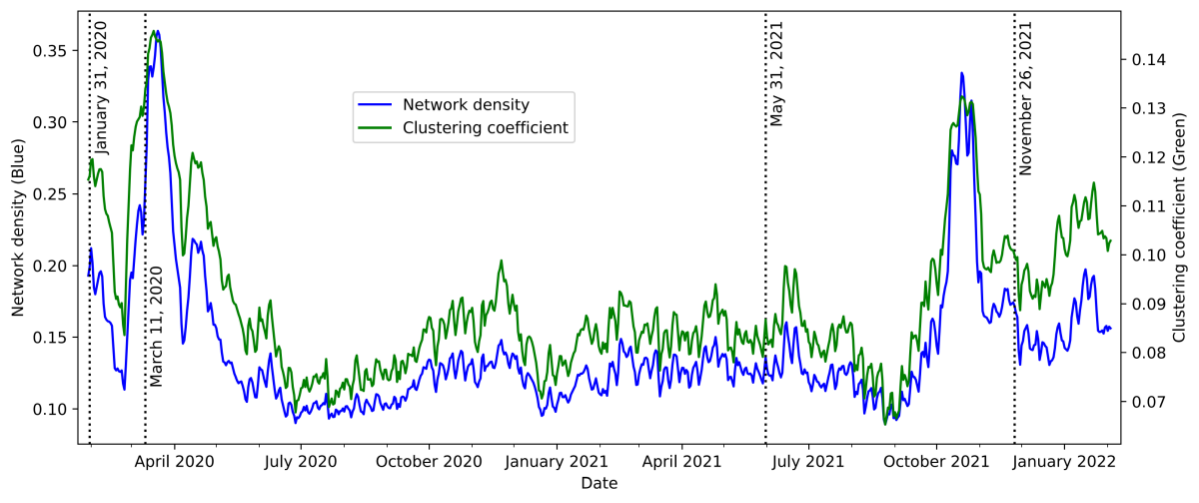
Network density and global clustering coefficients of the merged Google Trends network over time. The occurrences of significant events are marked with dotted lines. The 4 significant events marked are as follows: (1) COVID-19 was declared a “Public Health Emergency of International Concern” (on January 31, 2020), (2) COVID-19 was declared a pandemic (on March 11, 2020), (3) the Delta variant was named (on April 31, 2021), and (4) the Omicron variant was named (on November 26, 2021).

To study the network macroscopically, we computed 2 network statistics over time—the network density and the global clustering coefficient—by using a 30-day rolling window. The network density is a measure of the node connectedness of a network, whereas the global clustering coefficient represents the ratio of the number of established connections (edges) to the number of possible connections. The higher their values, the more interconnected the network nodes are. Figure 6 presents a time series of the GT network density (blue line) and a time series of the GT network clustering coefficient (green line). In Figure 6, we also mark the 4 time points from Figure 5 to assess the relationships among the 2 network statistics, network density and global clustering coefficient, and the propagation of COVID-19. In the very early stage of COVID-19, on January 31, 2020, both the network density and global clustering coefficient were in the middle level of approximately 0.2 for the network density and 0.12 for the global clustering coefficient (the maximum value is 1.0). Probably because of the warnings of an emerging public health concern and because some keywords are not directly related to the pandemic, the search interest intensity was already quite high in January 2020. Both network statistics climbed to a peak of approximately 0.35, which alerted people to the severity of the disease’s spread, thus inducing more search interest for the keywords at the same time. Subsequently, the network density remained at a relatively low level of approximately 0.15, even when the Delta variant was named on May 31, 2021. This reflects a relatively lower attention to the pandemic among the general public owing to people’s adaptation to and fatigue from the long-term antipandemic living environment. What is particularly alarming is the occurrence of another peak in the 2 network statistics during October to early November 2021, before the Omicron variant was named. The GT network density and global clustering coefficients show a substantial increase in the public’s interest in searching for keywords, probably indicating growing public awareness about and panic toward the next phase of the pandemic. Not long after that, in early 2022, the Omicron variant had a great impact on many countries.

### Comparing Network Statistics and SRSs Using DTW

**Figure 7:**
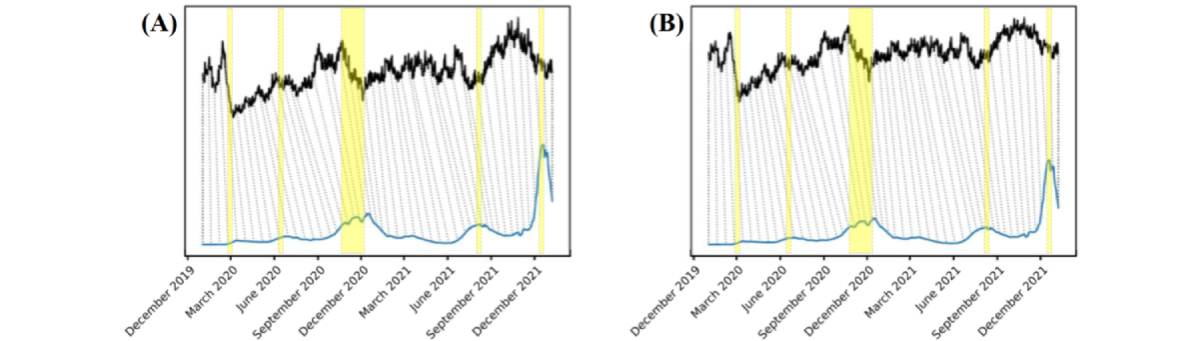
The 2-way dynamic time warping diagrams of the confirmed new COVID-19 cases in the United States (blue line) and the network statistics (black line) of the selected keywords in Google Trends: (A) network density trend versus confirmed new cases in the United States and (B) trend of the global clustering coefficient versus confirmed new cases in the United States. Yellow highlighted areas mark the 5 wave peaks observed in confirmed new cases in the United States.

**Figure 8:**
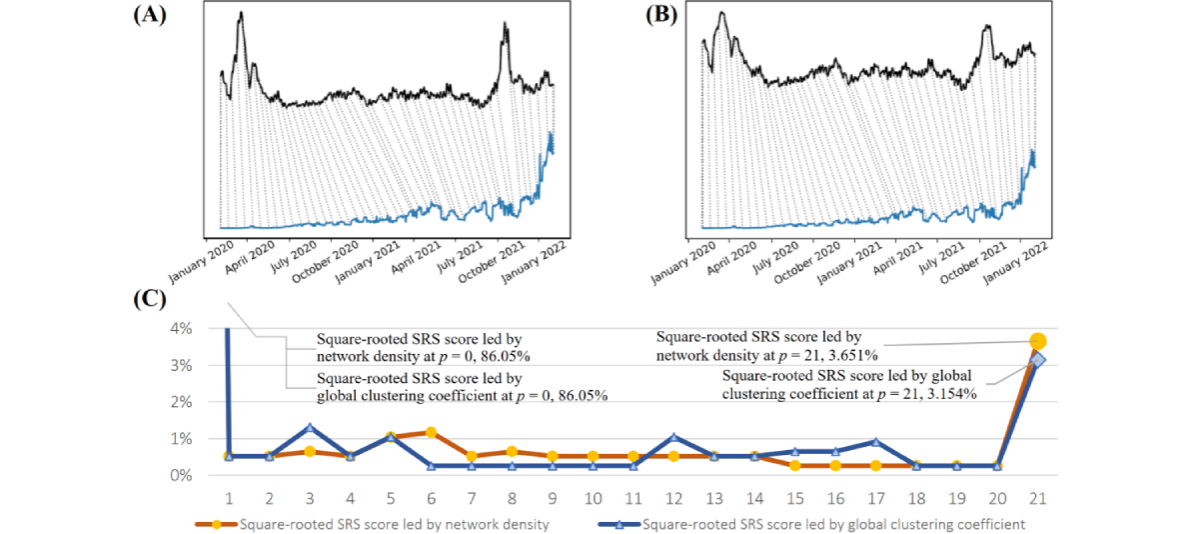
An illustrative evaluation of dynamic time warping (DTW) connections of the severity risk scores (SRSs) of the COVID-19 pandemic in the United States and the network statistics. Top: the 2-way DTW diagrams of the SRSs of the COVID-19 pandemic in the United States (blue line) and the network statistics (black line) of the selected keywords in Google Trends: (A) network density trend versus SRSs and (B) trend of global clustering coefficients versus SRSs. Bottom: (C) The summary plot of the time shifts (p) in each connection in (A), drawn with orange line, and (B), drawn with blue line. The data points at time shift *p* = 0 are muted in the plot.

Figures 7 and 8 illustrate the association between network statistics and the COVID-19 pandemic propagation in multiple directions. As mentioned in the *MSVs and COVID-19 Waves* section, the waves of COVID-19 cases represent the progression of the pandemic. As Figure 7 shows, there was an upward trend and peak in the dynamic network before every peak of the COVID-19 pandemic wave (highlighted in yellow). This shows that the dynamic networks of related keywords were capable of providing an early signal of not only the first outbreak but all 5 outbreaks throughout the COVID-19 pandemic (as shown in Figures 7A and 7B).

We further examined the predictive power of the dynamic network for the coronavirus-related keywords using the DTW algorithm [38,39]. By evaluating the similarity between the network and the newly confirmed cases in the United States, the resulting paths (the dotted lines) connect the 2 series of data and show an asymmetric step pattern. The consistently backslashed direction of the dotted lines suggests the lag-lead relationship between the dynamic network and the variation in the daily confirmed cases.

To evaluate the pandemic predictive capability of the dynamic network among the selected keywords in multiple layers, we also computed the DTW scores between the network and SRSs). The SRS, adopted from the studies by So et al [37,50], is regarded as a systematic measure that assesses the risk of the COVID-19 outbreak worldwide. A higher SRS represents a stronger signal, indicating that the global pandemic is uncontrolled. The DTW diagram in Figure 8 shows the lead-lag relationship of the SRSs throughout the entire observation period of the COVID-19 pandemic. These findings indicate that the dynamic network of related search keywords can predict the severity of the global risks of a pandemic. If regional search data are used, the predictability of the regional pandemic risk is also possible.

### Quantify the Asynchronous Movement of Statistics

We further quantify our observations in the 2-way DTW plots in Figures 8A and 8B by tabulating the portion of different time shift values, which shows the equivalently compelling result. As shown in Figure 8C, most of the connections between the SRS series and the network statistics series were on days 0 and 21.

We are not particularly interested in non–time-shifted DTW connections. Owing to the design of the DTW algorithm, the start and end of the pair of latent variables and observations will often warp to each other one-on-one, without any time shift, resulting in a nontrivial portion of the connections between any 2 time series defaults to *P*= 0 (ie, no time shift). Thus, we can safely assume that there is an insignificant amount of information in these connections, and we also masked the corresponding points in Figure 8C.

In contrast, the substantial portion of 21-day-forward-shifted connections offers an interesting insight. As many of the DTW connections detected have some form of forward time shift, the connections indicate that the movement of summary network statistics has a certain leading effect on the COVID-19 observed statistics. On the basis of our visual inspection and the DTW metric, the potential of summary network statistics in predicting real-life COVID-19 events is quite evident.

### Dynamic Property of the MSV Network in the Pandemic Progression

**Figure 9:**
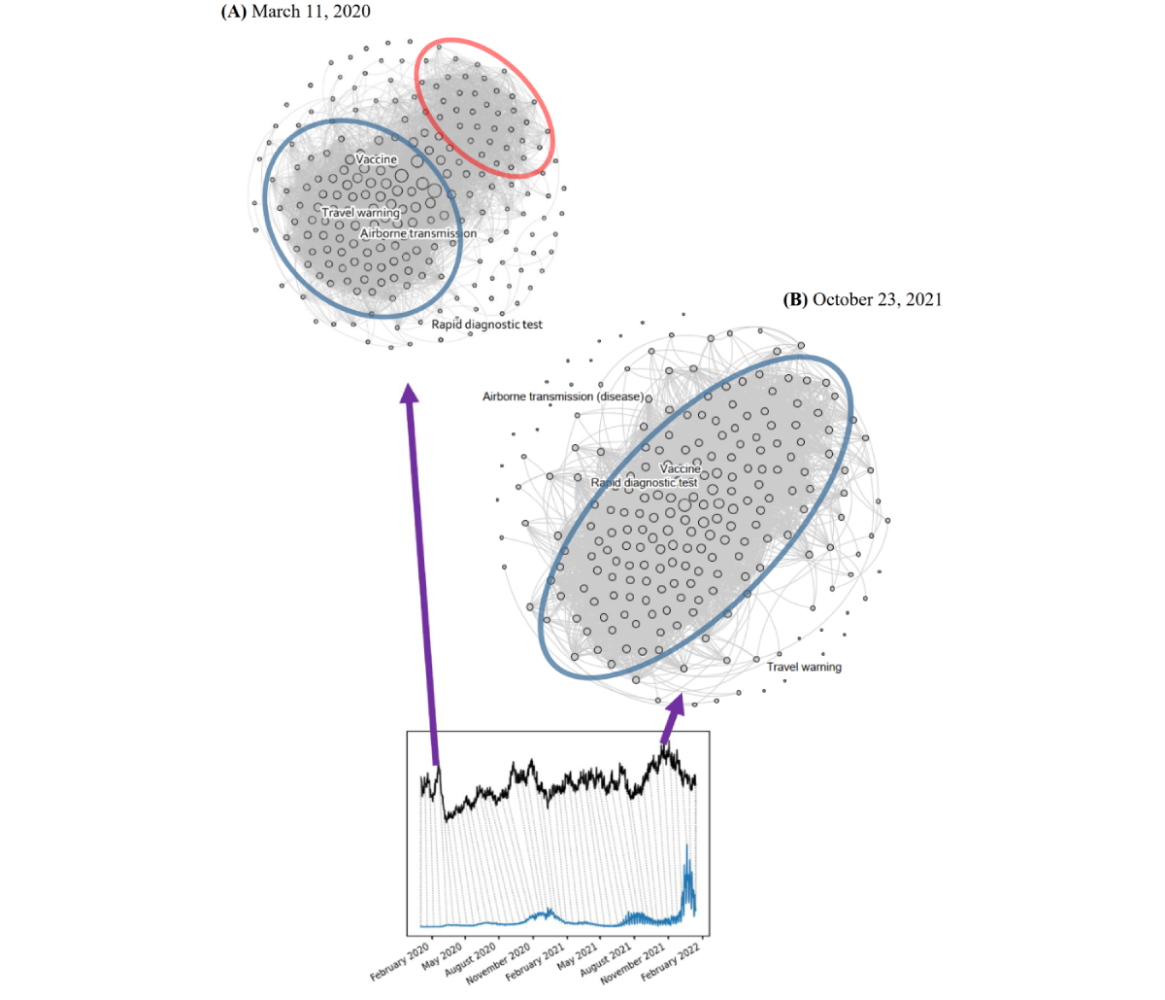
Visualization of the network construction using selected dates: (A) snapshot on March 11, 2020, and (B) snapshot on October 23, 2021. Highlighted nodes: search queries “rapid diagnostic test,” “vaccine,” “airborne transmission,” and “travel warning.” Large red and blue circles: observable clusters.

In addition to the magnitude of the dynamic network, we explored the pattern alterations during the progression of the pandemic. In Figure 9, the construction of the networks on March 11, 2020, and October 23, 2021, are depicted, where the MSV network peaks are observable before the first and the fifth COVID-19 outbreaks, respectively. In addition to network density, the global clustering coefficients for these 2 dates also form peaks, indicating high clustering coefficients in the network of the selected coronavirus-related keywords during those periods.

On March 11, 2020, the dense network was represented by noticeable clusters in Figure 9A, although a small separation was found between the 2 clusters. Although the nodes of search queries “Travel warning,” “Airborne transmission,” and “Vaccine” are connected within the cluster, the nodes for “rapid diagnostic test” are located farther away from it.

Comparatively, we observe a single large cluster in Figure 9B, which shows a generally high connectedness among the coronavirus-related keywords. Moreover, the indicated node “rapid diagnostic test” has shifted into the main cluster with another node, “vaccine.” This may reflect the increasing importance and availability of effective rapid diagnostic tests for COVID-19 worldwide in the latter phase of the pandemic’s progression. Conversely, the nodes “Travel warning” and “Airborne transmission” are located outside the main cluster, as shown in Figure 9B. This change in their position may imply a reduced focus in the community owing to a relatively sufficient understanding of COVID-19 transmission and control. This finding suggests that the proposed approach to a dynamic MSV network is a possible infoveillance application that would allow nonstationary patterns of search interests during the progression of a pandemic.

**Figure 10:**
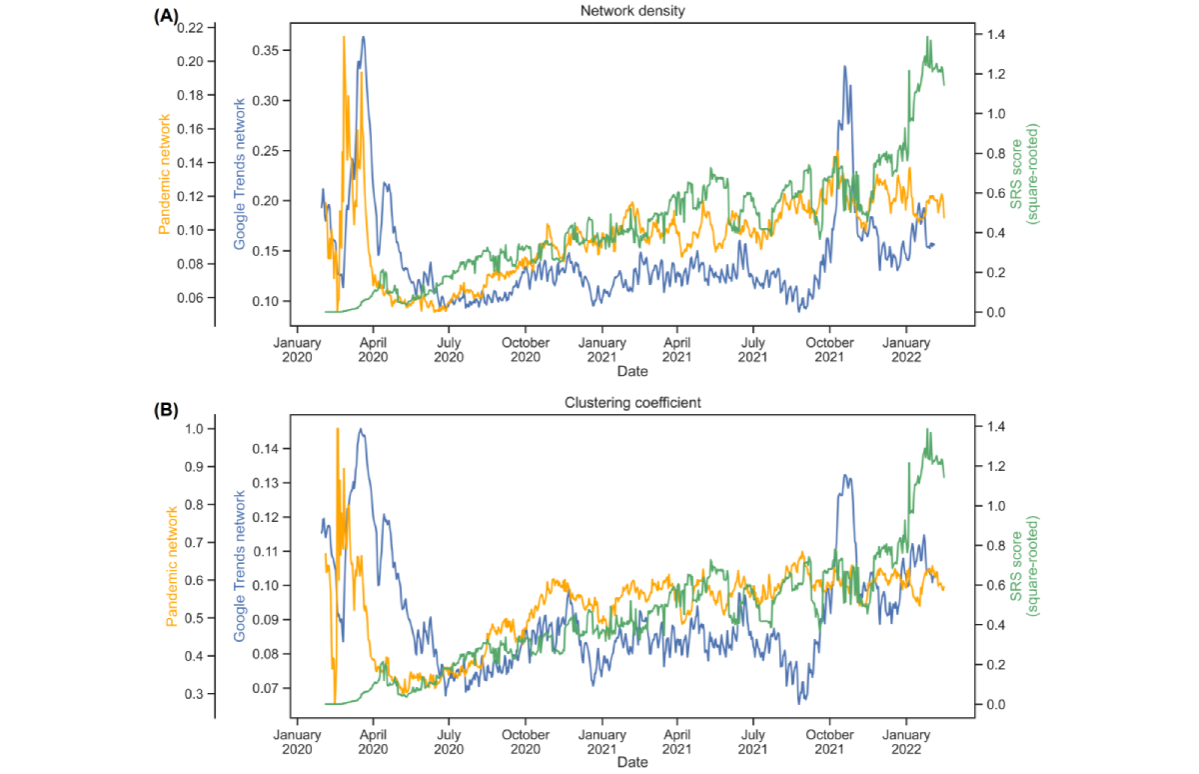
Two network statistics of the merged Google Trends (GT) network and the pandemic network, compared against the square roots of the severity risk scores (SRSs): (A) comparison with the GT network density and (B) comparison with the GT global clustering coefficient.

We also included the square root of the SRS [37,50] in Figure 10 for comparison. The pandemic network statistics showed an upward trend that aligned with the variations in SRSs. The GT network statistics even provided a sensible signal in late 2021, alerting to the latter peak of SRS, which probably reflected the notable impact of the Omicron variant worldwide.

## Discussion

### Principal Findings

This study introduced an innovative method for handling GT search data in research analysis, including an algorithm for constructing an MSV series and a dynamic GT network.

The original data extracted from GT have several limitations that affected the analysis in this study. First, the search volume data available in GT are normalized to the specified time and region [33]. The time series data of “interest over time” of a particular keyword only indicate the search interest relative to the highest point for a defined region and period. The resulting search data were also rescaled as integers ranging from 0 to 100 [33]. The normalization and rounding processes may lead to data inaccuracy in representing the search interest. In addition, GT restricts the availability of data over a long period. When one requests search data over a long duration, only weekly or monthly data will be returned, rather than daily data [25]. This restriction greatly reduces the resolution of the search data and affects further investigation [31,51]. Therefore, this study introduced a merged algorithm that helps recover the resolution and accuracy of the search volume data extracted from GT over long observation periods. The suggested MSVs can broaden the potential value of GT data in long-term surveillance of health phenomena.

### Strengths and Limitations

Using our novel merged algorithm, we observed similar phenomena in search volumes, which have been suggested in previous work, such as certain search terms correlated with COVID-19–confirmed cases [9], and there were increasing or decreasing searches for some keywords before major COVID-19 events [51]. However, unlike other previous infoveillance studies using GT [9,15-21,23-31,52-54], which often have to either sacrifice data resolution or data time horizon, we are able to use higher-resolution data with shorter delays for prolonged periods.

In addition to the merged algorithm, we demonstrated the potential of network analysis as a useful tool for infodemiology and infoveillance. Network analysis offers not only an integrated perspective of multiple time series but also cross-sectional visualization. In this study, we illustrated the cross-sectional network constructions at different timestamps in Figure 5 to investigate the clustering effect and associations of various keywords at different moments. We further presented an overall view of a temporal network via 2 summary statistics, namely, network density and global clustering coefficient, and revealed the lead-lag relationships between the official COVID-19 statistics and the summary statistics. The promising findings suggest that network analysis could be an important component for developing new predictive models.

Interestingly, the vaccine-related nodes (green nodes) in Figure 5 had a high degree of connectedness on all sampling days, even before any COVID-19 vaccine was approved for administration. It may be inferred that before the start of the pandemic, the public, health experts, and researchers searched for information about the coronavirus and its vaccines, resulting in a high number of correlations at the early stage, as shown in Figure 5A. Later, as the pandemic hit harder and the public began to understand more about COVID-19, people sought information more targeted at recovering from the COVID-19 pandemic, such as information on vaccinations and medications, as demonstrated in Figures 5C and 5D. In Figures 5C and 5D, disease-related and virus-related nodes drifted away from the cluster center, whereas nodes related to vaccines and pharmaceutical industry companies moved toward the center of the cluster.

Organizations such as government agencies and pharmaceutical companies were also on the radar of public awareness. At the beginning of the COVID-19 pandemic, members of the public understandably sought information on preventive measures and guidance from their government. As the pandemic continued, the public became familiar with social distancing and testing measures, and their attention shifted to vaccinations. As a result, the vaccines and the manufacturers of COVID-19 vaccines thus became part of the focal points, as illustrated in Figures 5C and 5D, where vaccine-related nodes (green) and nodes of pharmaceutical companies (purple) move toward the center of the cluster.

Our findings also revealed the capability of MSVs for the early detection of outbreak risk of emerging infectious diseases. Early signals are critical in controlling an emerging infection outbreak, as they allow policy makers and governments to impose rapid measures to curb the propagation of the disease. On the basis of our study findings, the MSVs of the generic keywords, such as the name of the infectious disease, symptoms, and transmission method, can provide an early signal of the risk of a potentially serious outbreak. This early detection may be related to self-diagnosis or information-seeking behaviors conducted via the internet, similar to the findings by Mangono et al [51]. Previous studies have raised the issue that a growing number of people will search the internet for health information about emerging diseases and their bodily changes and will make self-diagnoses [53] and seek preventive measures [23]. This causes the popularity of the related keywords to increase rapidly before an actual outbreak. In contrast, public transportation is considered as a factor in the spread of infectious diseases during the early stage of a pandemic. Therefore, the related keywords (eg, “travel warning” and “public transport” in Figures 2D and 2E) could be an indicator of citizens’ mobility intentions and provide useful information for predicting the spread of a pandemic.

In addition to discovering the early detection capability, we also revealed that different related keywords could have increased in popularity in search engines based on their roles at different stages of pandemic development. In the 5 observable waves of the COVID-19 pandemic in the United States since January 2020, each wave consisted of unique epidemiologic characteristics and caused the US government to take diverse actions to respond to the outbreaks [55,56]. Unlike other chronic diseases, the continual evolution of a pandemic and policy making may drastically vary the association between the public’s search interests and the progression of a disease of concern. Lu and Reis [19] also elaborated that the association between some search keywords and a disease’s progression may not be stable and persistent in the later waves of an outbreak because of increased public education about the particular disease.

By aligning the trends of MSVs with the COVID-19 pandemic waves, we discovered the dynamic property of multiple search queries when associated with the pandemic’s progression. This dynamic search pattern may be related to a shift in the public’s search interests regarding the same aspect of the pandemic. For example, personal hygiene was a crucial precautionary measure promoted during the first wave of the COVID-19 outbreak [54]. With appropriate health education and promotion, the public then developed a fundamental understanding of the measures, such as handwashing and mask wearing. With the 3 waves of COVID-19, people raised the demand for vaccines as an effective precaution against the disease [52]. As can be seen in our findings (Figures 2F and 2G), there was a shift of public search interests in precautionary measures from “hygiene” to “vaccine” after the third wave. Similarly, people with COVID-19 symptoms apparently considered “emergency medical service” during the early phases of the pandemic [57], but with the available “rapid diagnostic test” in 2021, those with symptoms tended to conduct the rapid tests before seeking medical services [58]. This yielded an observable shift in public search interests in diagnostic procedures from “emergency medical service” to “rapid diagnostic test” during the third wave. The shifting search interest may have been owing to changes in information-seeking behaviors before public policy implementation [51].

As the dynamic relationship between the internet data and the pandemic trends in the long run limited the potential of the conventional predictive models using search volumes as predictors [31], we mitigated this problem by considering the connectedness of the keywords, which could reflect the actual linkages of public concerns and human information-seeking behaviors. In this study, we provided an innovative application of network statistics that quantified the connectedness of MSV data in processing the prediction. Although multiple COVID-19 variants occurred that varied the pandemic’s development and the corresponding implementation of health measures [54], the resulting network trends could still be longitudinally associated with the 5 COVID-19 waves in the United States. By using DTW, the dynamic network of the MSVs was further justified as a leader of the trend of US-confirmed cases and of the severity of the global pandemic risk. Although previous studies revealed that linkages and patterns existed among the search queries specific to the pandemic [19,51], our proposed method took the nonstationary pattern among the relevant search queries into account during pandemic predictions. The method was able to demonstrate that the network of the merged GT volumes can be considered as a new form of official web-based data, not only associated with the pandemic but also capable of predicting its development.

This study’s approach using network analysis is innovative in the field of health and epidemiology research. Our early ground research provided a comprehensive empirical foundation for applying network statistics to investigate diverse pandemic-related issues, including infection topology [37], travel restrictions [43,47,49], and financial impacts [36,45,48]. In particular, pandemic networks using COVID-19 confirmed cases have proved their capability in tracking and providing early warnings of infection outbreaks [44,46]. We adapted the construction of a pandemic network to an infodemiological setting by investigating the dynamic GT network. In Figure 10, we compare our GT network statistics with the corresponding pandemic network statistics proposed in multiple relevant studies [36,37,44,46,47,49]. The pandemic network density and global clustering coefficients were shown to provide early warning signals of the COVID-19 pandemic [36,37,43]. Interestingly, both the GT network density and global clustering coefficients behaved similarly with the pandemic network statistics at the early stage of COVID-19 progression. On the basis of this comparison, we anticipate that both sets of network statistics can convey and deliver useful measures for pandemic risk and progression. As search data are considered to be complementary infoveillance measures, our proposed methodology of GT network analysis provides an important scientific discovery of the potential usefulness of search volume data in infodemiological research.

In addition to data analysis, the importance of keyword selection in infodemiology research has also been emphasized in the literature [59]. The procedure of excluding noisy queries is known to be important in ensuring the validity of research findings. However, there is a lack of a suitable framework for research on internet searching patterns during a pandemic. To prevent a stringent keyword selection by excluding potential keywords, we adopted a novel keyword selection approach based on relatively explicit data for “related topics” in GT. Although this approach helped us discover the keywords relevant to the pandemic and facilitated our interpretation of the findings, future studies in infodemiology could also adopt this approach to strengthen their selection of search queries.

We realize that our network consisted of regional topics associated with countries that have significantly higher than average internet coverage, such as Switzerland (internet penetration rate [IPR]: 98%), the United Kingdom (IPR: 98%), Japan (IPR: 94%), and the United States (IPR: 92%) [7]. Furthermore, IPR is skewed by the level of development of a country. However, because the proportion of those topics is as large as other nonregional terms, it is safe to assume that the network highlighted the interests of internet users in those regions, but the impact of the regional topics was nonsignificant and marginal.

Furthermore, we intentionally included most of the keywords we obtained from GT, as our research focuses on the worldwide public behavioral pattern during the COVID-19 epidemic. Despite the seeming irrelevance of some keywords, all the keywords collectively indicate the information the public received, with various levels of trustworthiness. Although only keywords for GT categories associated with regional effects were excluded in our study, future studies can examine the determined GT categories and exclude the keywords that are pathologically, psychosocially, or epidemiologically unrelated to their specific health concerns. In addition to the time and labor involved, researchers should consider the objectivity and validity of manually including or excluding certain keywords to develop the most rigorous data sets in their research.

### Conclusions

As the growth of the epidemic in the United States generally relates to the transmission across multiple countries, our research investigates the linkage between worldwide public behavioral patterns and the COVID-19 pandemic in the United States. Future studies should further investigate the association between regional GT data and noncommunicable diseases.

Although we did not focus on investigating the spread of misinformation during COVID-19, research has shown that receiving accurate and inaccurate information is inversely correlated with individuals’ compliance with COVID-19 health guidelines [60-63]. Our data also show that the public may actively seek information on misinformation, as evidenced by the noninsignificant search interest in the term “hydroxychloroquine” (Textbox 1). Owing to the nature of GT, we cannot confirm whether the users were searching for misinformation out of their beliefs in certain misinformation, the inclusion of users’ interest in the information of different trustworthiness [60-62] and users’ interest in those who propagate said information [40] is critical to a comprehensive infoveillance instrument.

To the best of our knowledge, this study is the first of its kind to use a rolling window ensemble method [45,64] to recover data resolution in GT. This is also the first study to investigate the network statistics of GT data for predicting the development of a pandemic. The results also provide insights for researchers in further investigating the patterns of web-based searching behaviors, which is an important focus in infodemiological research. With the ongoing rapid growth of internet use, people will rely increasingly more on web-based information for decision-making. As the search volume data are freely available on the GT platform, it is expected that GT will become more common as a complementary, free, and timely data resource for traditional surveillance methods of health-related events. Future studies can focus on expanding the dynamic networks of search keywords to investigate noncommunicable diseases, health-related behaviors, and misinformation on the internet.

## Abbreviations

DTW: dynamic time warping
GT: Google Trends
IPR: internet penetration rate
MSV: merged search volume
RSV: relative search volume
RT: related topic
SRS: severity risk score

## Data Availability

The source code and data sets generated during this study are available in the Zenodo repository [65].
